# Feasibility of preoperative chemotherapy for locally advanced, operable colon cancer: the pilot phase of a randomised controlled trial

**DOI:** 10.1016/S1470-2045(12)70348-0

**Published:** 2012-11

**Authors:** FOxTROT Collaborative Group

## Abstract

**Background:**

Preoperative (neoadjuvant) chemotherapy and radiotherapy are more effective than similar postoperative treatment for oesophageal, gastric, and rectal cancers, perhaps because of more effective micrometastasis eradication and reduced risk of incomplete excision and tumour cell shedding during surgery. The FOxTROT trial aims to investigate the feasibility, safety, and efficacy of preoperative chemotherapy for colon cancer.

**Methods:**

In the pilot stage of this randomised controlled trial, 150 patients with radiologically staged locally advanced (T3 with ≥5 mm invasion beyond the muscularis propria or T4) tumours from 35 UK centres were randomly assigned (2:1) to preoperative (three cycles of OxMdG [oxaliplatin 85 mg/m^2^, l-folinic acid 175 mg, fluorouracil 400 mg/m^2^ bolus, then 2400 mg/m^2^ by 46 h infusion] repeated at 2-weekly intervals followed by surgery and a further nine cycles of OxMdG) or standard postoperative chemotherapy (12 cycles of OxMdG). Patients with *KRAS* wild-type tumours were randomly assigned (1:1) to receive panitumumab (6 mg/kg; every 2 weeks with the first 6 weeks of chemotherapy) or not. Treatment allocation was through a central randomisation service using a minimised randomisation procedure including age, radiological T and N stage, site of tumour, and presence of defunctioning colostomy as stratification variables. Primary outcome measures of the pilot phase were feasibility, safety, and tolerance of preoperative therapy, and accuracy of radiological staging. Analysis was by intention to treat. This trial is registered, number ISRCTN 87163246.

**Findings:**

96% (95 of 99) of patients started and 89% (85 of 95) completed preoperative chemotherapy with grade 3–4 gastrointestinal toxicity in 7% (seven of 94) of patients. All 99 tumours in the preoperative group were resected, with no significant differences in postoperative morbidity between the preoperative and control groups: 14% (14 of 99) versus 12% (six of 51) had complications prolonging hospital stay (p=0·81). 98% (50 of 51) of postoperative chemotherapy patients had T3 or more advanced tumours confirmed at post-resection pathology compared with 91% (90 of 99) of patients following preoperative chemotherapy (p=0·10). Preoperative therapy resulted in significant downstaging of TNM5 compared with the postoperative group (p=0·04), including two pathological complete responses, apical node involvement (1% [one of 98] *vs* 20% [ten of 50], p<0·0001), resection margin involvement (4% [four of 99] *vs* 20% [ten of 50], p=0·002), and blinded centrally scored tumour regression grading: 31% (29 of 94) *vs* 2% (one of 46) moderate or greater regression (p=0·0001).

**Interpretation:**

Preoperative chemotherapy for radiologically staged, locally advanced operable primary colon cancer is feasible with acceptable toxicity and perioperative morbidity. Proceeding to the phase 3 trial, to establish whether the encouraging pathological responses seen with preoperative therapy translates into improved long-term oncological outcome, is appropriate.

**Funding:**

Cancer Research UK.

## Introduction

Preoperative (neoadjuvant) chemotherapy and radiotherapy are substantially more effective than similar postoperative therapy in oesophageal, gastric, and rectal cancer.^1^, ^2^, ^3^ Earlier treatment might be more effective at eradicating micrometastatic disease than the same treatment 3 months later,^4^, ^5^ the typical period between diagnosis and starting postoperative chemotherapy, particularly because surgery increases growth factor activity in the early postoperative period, promoting more rapid tumour progression.^6^, ^7^, ^8^

Shrinking of tumours before surgery might also reduce the frequency of tumour cell shedding during surgery^9^ and of incomplete excision.^2^, ^10^ Surgical resection margin involvement correlates strongly with locoregional recurrence,^11^ which can have a more aggressive phenotype^12^ and respond poorly to systemic therapy.^13^ Other potential advantages of preoperative therapy are to make minimum access surgery practicable, enabling earlier return to normal activity,^14^ and better tolerability than similar treatment after major surgery, hence allowing increased dose intensity.^3^ Assessment of response to preoperative chemotherapy might also be useful in guiding postoperative drug selection.

Although an attractive concept, preoperative chemotherapy has not, until now, been assessed in operable colon cancer because of concerns that, if tumour growth occurred during the preoperative treatment phase, this could result in bowel obstruction necessitating emergency surgery, an outcome associated with high morbidity and mortality. Another concern is that inaccurate radiological tumour staging might result in inappropriate chemotherapy for low-risk patients. However, with more effective regimens and advances in radiological staging,^15^ preoperative chemotherapy has become a promising option.

Response rates higher than 50% are consistently achieved in metastatic colorectal cancer with chemotherapy regimens combining fluoropyrimidines with irinotecan or oxaliplatin,^16^, ^17^ and even higher responses can be achieved—in *KRAS* wild-type tumours—by adding EGFR-targeted monoclonal antibodies, panitumumab or cetuximab, to combination chemotherapy.^17^, ^18^, ^19^, ^20^, ^21^, ^22^, ^23^, ^24^, ^25^, ^26^, ^27^, ^28^, ^29^, ^30^ The proportional improvements in tumour response rate with anti-EGFR monoclonal antibodies depend on treatment stage, but the absolute improvements are similar: 15% (15% *vs* 0·4%, p<0·0001)^18^, ^19^, ^20^ when used as single agents for patients who had not responded to standard chemotherapy, 18% (25% *vs* 7%, p<0·0001)^21^, ^22^, ^23^ when added to second-line irinotecan-based chemotherapy, and 9% (57% *vs* 48%, p<0·0001)^24^, ^25^, ^26^, ^27^, ^28^, ^29^, ^30^ when added to first-line chemotherapy (appendix). Efficacy of first-line anti-EGFR monoclonal antibodies appears similar when background chemotherapy is fluorouracil or capecitabine-based and with oxaliplatin or irinotecan.^31^ Thus, although EGFR-targeted therapies are ineffective, and might be harmful, in *KRAS* mutant tumours,^20^, ^21^, ^24^, ^27^ response of metastatic *KRAS* wild-type colonic tumours is clearly increased by adding anti-EGFR therapies to chemotherapy, suggesting potential benefits as an adjuvant to preoperative oxaliplatin-based chemotherapy in operable disease.

Moreover, tumours suitable for preoperative chemotherapy can now be accurately identified with a CT risk stratification algorithm based on the depth of tumour invasion beyond the muscularis propria.^15^ High-risk patients (stage T4 or T3 with ≥5 mm tumour invasion beyond muscularis propria) have a 53% 3-year recurrence-free survival compared with 87% for the good (T1/T2) or intermediate (T3 and <5 mm tumour invasion beyond muscularis propria) prognostic groups.

The FOxTROT (Fluoropyrimidine Oxaliplatin and Targeted Receptor Pre-Operative Therapy) trial was designed to assess whether 6 weeks of an effective combination chemotherapy regimen given preoperatively to patients with radiologically staged, locally advanced, but potentially resectable colon cancer improves disease-free survival, and whether the addition of an EGFR-targeted monoclonal antibody, panitumumab, to preoperative chemotherapy increases tumour shrinkage for patients with wild-type *KRAS* tumours. A feasibility phase, reported here, was incorporated to assess patient selection and recruitment, safety, and tumour response to preoperative treatment.

## Methods

### Patients

Eligible patients were 18 years or older with locally advanced (T4 or T3 with extramural depth ≥5 mm) adenocarcinoma of the colon, with staging determined preoperatively by either spiral or multidetector CT^15^ and for whom a 24-week course of oxaliplatin and fluoropyrimidine-based adjuvant chemotherapy would be judged appropriate. Patients were required to have adequate blood counts—haemoglobin greater than 100 g/L after transfusion and before surgery and chemotherapy, greater than 3·0×10^9^ white blood cells per L, and greater than 100×10^9^ platelets per L; adequate renal biochemistry with a glomerular filtration rate of greater than 50 mL per minute as calculated by the Wright or Cockroft formula or EDTA clearance of greater than 70 mL per minute; adequate hepatobiliary function with bilirubin less than 25 μmol per L; and serum magnesium levels within the normal range at trial entry. Written consent was obtained from patients, and ethics approval was obtained from the West Glasgow multicentre ethics committee (Glasgow, UK). The protocol is available online.

### Randomisation and masking

**Figure 1:**
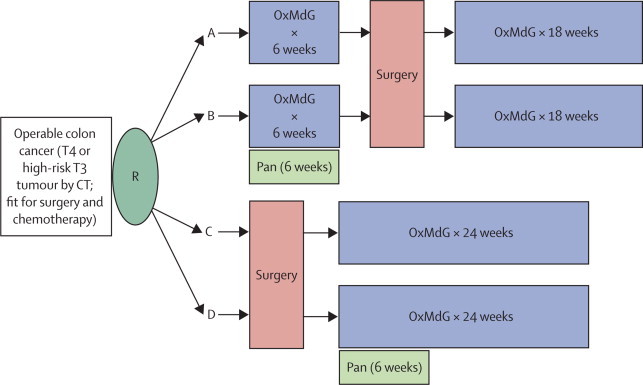
FOxTROT trial schema OxMdG=modified de Gramont chemotherapy. Pan=panitumumab. CT=computed tomography. R=randomisation.

Eligible patients were randomly assigned, in a 2:1 ratio, between preoperative plus postoperative and postoperative chemotherapy. Patients were also randomly assigned, in a 1:1 ratio, to receive panitumumab with the first 6 weeks of chemotherapy or not (figure 1). Patients were allocated to a treatment group by a telephone or web-based central randomisation service at the University of Birmingham Clinical Trials Unit (Birmingham, UK). A computerised minimised randomisation procedure was used to ensure a good balance between groups for age (<50, 50–59, 60–69, ≥70 years), radiological T-stage (T3, T4), radiological nodal status (Nx, N0, N1, N2), site of primary tumour, and defunctioning colostomy (yes, no). Treatment was open-label.

Soon after the start of FOxTROT, the poor effectiveness of EGFR monoclonal antibodies in tumours harbouring a *KRAS* mutation^18^, ^19^, ^20^ mandated introduction of preoperative *KRAS* testing of diagnostic biopsy samples, with only *KRAS* wild-type tumours eligible for the panitumumab randomisation. A two-stage consent and randomisation procedure was introduced. Patients were first invited to consent to molecular testing, then to randomisation for either preoperative plus postoperative chemotherapy or postoperative chemotherapy only. Only patients whose tumours tested as *KRAS* wild-type were entered in the panitumumab randomisation.

### Procedures

Chemotherapy was the standard UK modified de Gramont (OxMdG) regimen,^32^ consisting of cycles of oxaliplatin at 85 mg/m^2^ combined with l-folinic acid 175 mg/m^2^ plus fluorouracil 400 mg/m^2^ by intravenous bolus, followed by a 46 h infusion of 2400 mg/m^2^ through an indwelling line, repeated at 2-weekly intervals. Capecitabine could not be substituted for fluorouracil and folinic acid in this pilot study because of higher toxicity when combined with panitumumab.^17^ Dose reductions and delays of up to 4 weeks were allowed for reversible toxicity. Preoperative chemotherapy duration was only 6 weeks (three cycles of OxMdG) to minimise the risk of progression of chemoresistant tumours (15–20% of advanced metastatic colon cancers progress during 12 weeks of similar combination chemotherapy).^16^, ^17^, ^24^, ^26^ Surgery with curative intent was undertaken at least 3 weeks after completing preoperative therapy, to reduce perioperative morbidity,^2^ followed by a further 18 weeks (nine cycles) of OxMdG. CT scans were repeated before surgery in the preoperative group. For the patients who were not assigned to receive preoperative chemotherapy, postoperative chemotherapy duration was 24 weeks (12 cycles) of OxMdG. If allocated, panitumumab (6 mg/kg) was given by intravenous infusion at 2-weekly intervals during the first 6 weeks of chemotherapy (preoperative or postoperative).

The primary outcomes of the pilot phase were feasibility, safety, tolerance of preoperative therapy, and the accuracy of radiological staging. Other key outcomes were completion of planned surgery, perioperative morbidity, timely completion of preoperative *KRAS* testing, and downstaging of the resected tumour as measured by histopathological tumour diameter and stage. Tumour staging was standardised across centres, with training provided for participating specialist gastrointestinal radiologists and histopathologists. Pathological reports and tumour blocks were collected centrally with tumour regression grades scored masked to treatment allocation.

### Statistical analysis

The FOxTROT study aims to randomly assign at least 1050 patients to detect a 25% proportional reduction (roughly 8% absolute difference) in recurrence at 2 years (eg, 32% reduced to 24%) with 80% power at p<0·05. The prespecified sample size of 150 for the pilot phase was chosen pragmatically as a sufficient number to assess the potential rate of recruitment and any large differences in other primary outcomes. An independent steering committee advised whether to continue to the full study. Comparisons of preoperative versus postoperative chemotherapy were by intention to treat including all patients randomly assigned to treatment groups, ignoring panitumumab allocation, and using *t* tests to compare continuous variables, Mantel-Haenszel tests of association for ordinal variables, and SAS 9.2 statistical software.

The trial is registered, number ISRCTN 87163246.

### Role of the funding source

The funders of the study had no role in study design, data collection, data analysis, data interpretation, or writing of the report. All authors of the writing committee had full access to all the data in the study and had joint final responsibility for the decision to submit for publication.

## Results

Between May 15, 2008, and Sept 21, 2010, 150 patients from 35 UK centres were randomly assigned to receive either preoperative plus postoperative chemotherapy (n=99) or standard postoperative chemotherapy alone (n=51). Radiologically classified tumour characteristics were similarly distributed across treatment groups (table 1). Patient screening logs suggested that the main reason for clinicians not entering radiologically eligible patients was unsuitability for combination chemotherapy because of age or frailty. Patients were also excluded because of uncertainty about eligibility on CT staging, principally on whether depth of invasion beyond the muscularis propria was 5 mm or more. Premature scheduling of the operation date was also a common exclusion reason before the multidisciplinary teams and individual clinicians adapted to the new care pathway.

**Table 1:** Patient characteristics at baseline radiology

		**Preoperative plus postoperative chemotherapy (n=99)**	**Postoperative chemotherapy only (n=51)**
Age (years)			
	Median (IQR)	64 (59–68)	65 (56–69)
	Range	31–82	38–78
Age group (years)			
	<50	9 (9%)	4 (8%)
	50–59	16 (16%)	10 (20%)
	60–69	50 (51%)	26 (51%)
	≥70	24 (24%)	11 (22%)
Sex			
	Male	65 (66%)	32 (63%)
	Female	34 (34%)	19 (37%)
Colonic obstruction		3/99 (3%)	1/51 (2%)
WHO performance status			
	0	67 (68%)	34 (67%)
	1	30 (30%)	17 (33%)
	2	2 (2%)	0
Location			
	Caecum	23 (23%)	11 (22%)
	Ascending colon	18 (18%)	11 (22%)
	Hepatic flexure	5 (5%)	3 (6%)
	Transverse colon	7 (7%)	3 (6%)
	Splenic flexure	3 (3%)	1 (2%)
	Descending colon	3 (3%)	3 (6%)
	Sigmoid	32 (32%)	15 (29%)
	Rectosigmoid	8 (8%)	4 (8%)
Radiological T-stage			
	T3	69 (70%)	35 (69%)
	T4	30 (30%)	16 (31%)
Radiological N-stage			
	Nx	3 (3%)	1 (2%)
	N0	23 (23%)	12 (24%)
	N1	44 (45%)	22 (43%)
	N2	29 (29%)	16 (31%)
Extramural vascular invasion		57/98 (58%)^*^	31/51 (61%)
	Mean (SD)	12·9 (8·8)	15·5 (9·9)
	Range	1–50	5–50

One radiology form had missing extramural vascular invasion data.

138 of the 150 patients were eligible for *KRAS* testing to establish eligibility for the panitumumab randomisation: two were entered before *KRAS* testing was introduced, six were entered while the panitumumab randomisation was suspended, and four were from centres not taking part in the panitumumab randomisation (appendix). Biopsy samples were obtained for 98% (135 of 138) of these patients and 96% (130 of 135) were successfully tested. 72% (95 of 132 with known *KRAS* status, including the two patients retrospectively tested) were *KRAS* wild-type of whom 90 were randomly assigned to either panitumumab or control, with four *KRAS* wild-type results arriving too late for panitumumab randomisation and one patient declining randomisation. 46 (31% of all 150 patients) were allocated panitumumab. The median time from consent to *KRAS* test result and panitumumab randomisation was 9 days (IQR 7–12).

**Figure 2:**
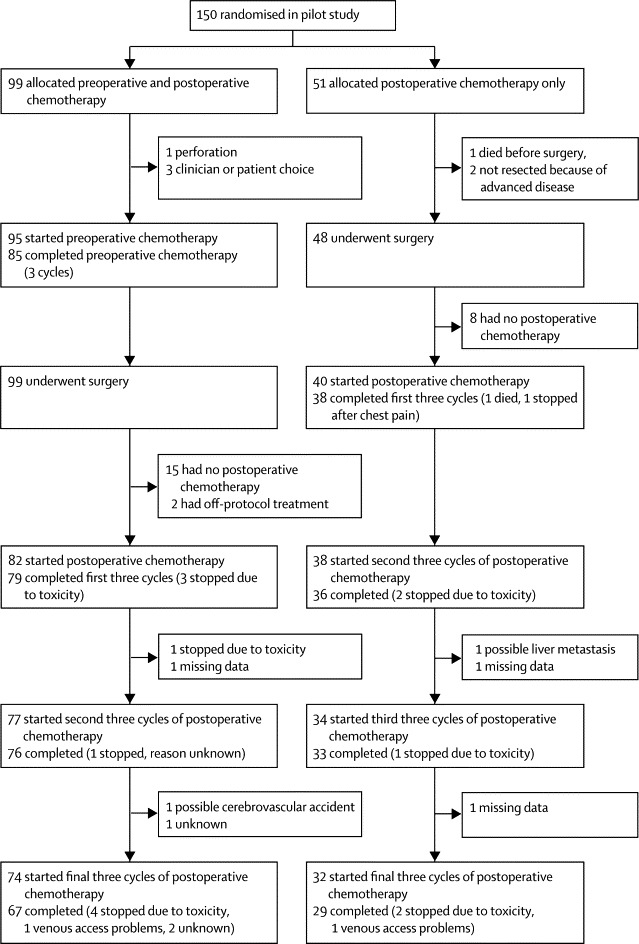
FOxTROT patient pathway

Of 99 patients allocated preoperative chemotherapy, 95 (96%) started treatment as planned with one proceeding directly to surgery after diagnosis of a localised perforation and three because of patient or clinician's choice (figure 2). The mean delay from randomisation to start of chemotherapy was 13 (SD 6) days. 89% (85 of 95) of patients starting preoperative treatment completed the 6-week course with nine stopping early because of toxicity and one withdrawing to off-trial bevacizumab treatment. All 99 patients underwent resectional surgery with a mean time to surgery from start of chemotherapy of 61 (SD 15) days. 83% (82 of 99) continued with postoperative OxMdG chemotherapy. 15 patients had no postoperative chemotherapy; five patients (including three who had no preoperative chemotherapy) did not have postoperative chemotherapy because of low-risk pathology, five because of previous adverse events (four because of toxicity of preoperative chemotherapy, one because of surgical morbidity), three refused, one had metastatic disease, and one died in the postoperative period. Additionally, two had off-protocol treatment (one patient had a different chemotherapy regimen, and one was treated with bevacizumab). 96% (79 of 82) of those starting completed the first 6 weeks (three stopped because of toxicity) and 82% (67 of 82) completed all 18 weeks of postoperative treatment. Toxicity was the most common reason for stopping treatment, with nine patients stopping at some point during postoperative chemotherapy (four during or after the first three cycles of postoperative chemotherapy, one after the second three cycles, and four during or after the final three cycles).

Of 51 patients allocated postoperative chemotherapy, 94% (48 of 51) had resectional surgery with one dying beforehand and two who were not resected because of inoperable peritoneal spread detected at surgery (figure 2). 78% (40 of 51) started postoperative chemotherapy with 11 not having postoperative chemotherapy because of low-risk pathology (seven) or dying beforehand (four). 95% (38 of 40) of those starting completed the first 6 weeks and 72% (29 of 40) completed all 24 weeks of chemotherapy. Toxicity caused six patients to discontinue chemotherapy.

Thus, a higher proportion of patients started preoperative than postoperative chemotherapy (96% [95 of 99] *vs* 78% [40 of 51]; p=0·001), and chemotherapy completion rates were also higher in the preoperative therapy group with 68% (67 of 99) of those allocated pre plus postoperative chemotherapy completing 24 weeks of treatment compared with 57% (29 of 51) of postoperative chemotherapy patients (p=0·19).

Accuracy of radiological staging was assessed by pathological examination of resected tumours from the postoperative chemotherapy only group. 86% (43 of 50 [including two that were unresected]) of tumours had adverse features (inoperable tumour, positive lymph nodes, extramural vascular invasion [EMVI], or depth of invasion ≥5 mm) on pathological examination, indicative of a greater than 50% recurrence risk at 3 years. Radiology accurately identified invasion of the muscularis propria, with only 2% (one of 51) of patients in the postoperative group having a pathological T2 (pT2) tumour; a further 12% (six of 51) had pT3 tumours without additional risk factors. Radiology was less accurate in discriminating between T3 and T4 stage, with 47% (eight of 17) of pT4 tumours also T4 on radiological assessment and 50% (8 of 16) of those T4 on radiology were pT3 on pathological assessment. Radiological assessment was more sensitive in detecting nodal spread with 83% (20 of 24) of pathological node-positive patients also node-positive on radiological assessment. However, specificity was low with radiological staging tending to overestimate tumour spread: 44% (16 of 36) of those node-positive on radiology were node-negative on pathological assessment. Similarly, 52% (14 of 27) of those with extramural vascular invasion on radiology were EMVI-negative on pathological assessment.

One patient in each group needed acute surgery because of incipient obstruction, with both proceeding to resectional surgery. No significant differences were seen in complication rates (table 2) or median time to hospital discharge (preoperative plus postoperative chemotherapy group: 7 days, IQR 5–10; postoperative only: 6 days, 3–8; p=0·18) although there was a greater proportion of wound infections in the preoperative group (table 2). The proportions of patients with complications that prolonged hospital stay, or had procedures that resulted in a stoma or required further adominal surgery for anastomotic complications, were much the same for the two groups (table 2). The median delay after surgery to starting chemotherapy was 47 days (IQR 40–55) for patients allocated preoperative chemotherapy and 53 days (44–57) for patients allocated postoperative chemotherapy.

**Table 2:** Perioperative complications in the preoperative plus postoperative chemotherapy group compared with the postoperative chemotherapy group

	**Preoperative plus postoperative chemotherapy(n=99)**	**Postoperative chemotherapy only (n=51)**	**p value**
Anastomotic leak	5 (5%)	2 (4%)	0·77
Wound infection with or without intra-abdominal abscess*	13 (13%)	4 (8%)	0·34
Bronchopneumonia	2 (2%)	0	0·31
Deep vein thrombosis	2 (2%)	0	0·31
Rash	3 (3%)	0	0·21
Neutropenia	1 (1%)	0	0·47
Death	0	1 (2%)	0·16
Other	12 (12%)	6 (12%)	0·72
Complication prolonging hospital stay	14 (14%)	6 (12%)	0·81
Procedure resulting in a stoma	12 (12%)	5 (10%)	0·66
Further abdominal surgery needed	4 (4%)	2 (4%)	0·96

* All three patients with intra-abdominal abscess also had wound infection recorded.

Of those receiving chemotherapy, toxicity was similar between groups in the first 6 weeks and in subsequent treatment (appendix). 34% (32 of 94) of preoperative and 31% (12 of 39) of postoperative chemotherapy patients had grade 3 or worse toxicity in their first 6 weeks of chemotherapy (p=0·72). 7% (seven of 94) of preoperative and 10% (four of 39) of postoperative chemotherapy patients had grade 3 or worse gastrointestinal toxicity; 11% (nine of 85) versus 21% (eight of 38) required dose reductions (p=0·12) and 11% (ten of 95) versus 5% (two of 40) did not complete their first 6 weeks of chemotherapy (p=0·31). Only one patient had surgery delayed, for 2 weeks, because of toxicity of preoperative chemotherapy—grade 4 neutropenia. For all 24 weeks of chemotherapy, 49% of patients (47 of 95) in the preoperative chemotherapy group and 51% of patients (20 of 39) in the postoperative group had any grade 3 or higher adverse events, with the most common being haematological (29% [28 of 95] and 28% [11 of 39]) and gastrointestinal (19% [18 of 95] and 21% [eight of 39]) adverse events (appendix).

Significant differences favouring preoperative therapy were seen in apical node involvement in the resected specimens (one of 98 *vs* ten of 50; p<0·0001), TNM5 staging (p=0·04), resection margin involvement (four of 99 *vs* ten of 50; p=0·002), and retroperitoneal margin involvement (five of 94 *vs* eight of 44; p=0·016; table 3). 31% of (29 of 94) tumours in the preoperative group with blinded centrally scored regression grades showed moderate to complete regression as compared with 2% (one of 46) in the control series (p=0·0001). There were reductions in tumour diameter, tumour thickness, and depth of spread beyond the muscularis propria in the preoperative group compared with controls. There were two complete pathological responses and seven T2 tumours in the preoperative group, with only one T2 tumour in the control group. However, downstaging as assessed by T stage was not significant (table 3). The proportion of tumours with involved lymph nodes was lower (40% [39 of 98] *vs* 52% [26 of 50], test for association p=0·039) in the preoperative chemotherapy group, with the number of lymph nodes identified for histology assessment being similar between groups.

**Table 3:** Tumour characteristics on pathological examination

		**Preoperative and postoperative chemotherapy group (n=99)**	Postoperative chemotherapy only group (n=50)*	**p value**
Resection margins				
	R0–complete	95 (96%)	40 (80%)	0·002
	R1–incomplete/R2	4 (4%)	10 (20%)	..
Mean distance to nearer margin (mm)		68·4 (56·6); n=97	70·9 (59·9); n=46	0·81
Maximum tumour thickness (mm)		18·5 (12·1); n=61	24·4 (16·7); n=33	0·08
Maximum tumour diameter (mm)		49·6 (45·4); n=93	62·2 (28·0); n=46	0·05
Distance to retroperitoneal margin (mm)		20·7 (16·1); n=65	19·7 (25·6); n=32	0·85
Maximum spread beyond muscularis propria (mm)		6·9 (6·4); n=89	8·7 (7·4); n=43	0·14
T stage TNM5				
	T0 (no tumour)	2	0	MH=0·16; MH combining T0/1/2 and T4=0·20
	T1 (invades submucosa)	0	0	..
	T2 (invades muscularis propria)	7	1	..
	T3 (invades through muscularis propria)	60	30	..
	T4 (penetrates to peritoneum)	17	11	..
	T4 (invades adjacent organs)	13	8	..
N stage TNM5				
	Nx	1	0	..
	N0	59	24	MH=0·039
	N1 (1–3 nodes)	24	10	..
	N2 (≥4 nodes)	15	16	..
Lymph nodes examined				
	0–5	2	0	MH=0·25
	6–11	6	2	..
	12–20	40	16	..
	21–30	33	19	..
	31–40	12	8	..
	≥40	6	3	..
	Median	21 (15–27)	22 (16–30)	0·20
	Apical nodes positive	1/98	10/50	<0·0001
	Extramural vascular invasion	34/97	24/48	0·085
American (TNM5) staging				
	No tumour	2	0	MH=0·04
	Stage 1	6	1	..
	Stage 2 (low risk)	17	6	..
	Stage 2 (high risk†)	35	17	..
	Stage 3	38	24	..
	Stage 4	1	2	..
Tumour regression grading				
	Complete response	2	0	MH=0·0004; any *vs* little/no regression MH=0·0001
	Marked regression	2	0	..
	Moderate regression	25	1	..
	Little/no regression	65	45	..

Data are n (%), mean (SD), or median (IQR).

* The two extra tumours are the two that were unresected. Macroscopic evaluation was used.

† Either T4, T3 with extramural vascular invasion, or T3 with ≥5 mm invasion of the muscularis propria. MH=Mantel-Haenszel test, TNM5=American Joint Committee on Cancer, fifth edn.

55% (11 of 20) of the tumours apparently downstaged between initial radiological assessment and pathological examination were also downstaged on radiology assessment after preoperative chemotherapy. None were upstaged. Conversely, 35% (six of 17) of the tumours apparently upstaged between initial radiological assessment and pathological examination were also upstaged on postchemotherapy radiology. None were downstaged. Reductions in the mean depth of spread beyond the muscularis propria (12·8 [SD 8·4] to 9·0 [7·9] mm; p=0·0022) and in the maximum tumour thickness (24·9 [12·2] to 19·0 [12·8] mm; p=0·0018) were also seen in the second radiological assessment compared with baseline.

## Discussion

FOxTROT is, to our knowledge, the first randomised trial to evaluate preoperative chemotherapy in primary colon cancer (panel). The pilot phase has provided clear evidence of downstaging with only 6 weeks of preoperative treatment. The significant reductions in apical node involvement, incomplete resections, and two pathological complete responses, are noteworthy since all have previously been shown to correlate with protracted disease-free survival.^11^ Tumour regression grading after preoperative chemotherapy also correlates with recurrence risk in other gastointestinal malignancies,^33^ and longer follow-up of the FOxTROT cohort should establish whether tumour regression grading is similarly prognostic in colon cancer.

Panel **Research in context**

**Systematic review**

We searched Medline and Embase for the MeSH and free terms: “colon” or “colonic” near “cancer”, “carcinoma”, “tumor”, “tumour”, or “neoplasm” and drug therapy or chemotherapy and neoadjuvant or preoperative combined with appropriate study design filter as described in section 6.4.11 of *The Cochrane Handbook of Systematic Reviews of Interventions*. No date or language restrictions were applied. No randomised trials comparing preoperative systemic chemotherapy with no chemotherapy in operable colon cancer were identified.

**Interpretation**

6 weeks of preoperative OxMdG chemotherapy for radiologically staged, locally advanced operable primary colon cancer is feasible with acceptable toxicity and perioperative morbidity and is associated with a significant pathological response. Further investigation of neoadjuvant chemotherapy of colon cancer is needed to evaluate long-term oncological outcome.

We have also shown that preoperative therapy is practicable and safe with no increase in surgical morbidity or mortality—a major focus of the feasibility study. With a 3-week delay after chemotherapy, surgery can be completed within 10 weeks from diagnosis. A small proportion (17%) of patients in the preoperative chemotherapy group had apparent progression between diagnostic radiology and post-resection pathology but, reassuringly, no patients developed incipient obstruction during the 6-week preoperative chemotherapy. A central review of CT scans is being undertaken to investigate whether the apparent upstaging was due to inaccurate initial radiological staging or true progression and will be reported separately. The absence of measurable adverse effects in terms of prolonged hospital stay, stoma formation, reoperation rates, and leak rates is, again, encouraging. A trend towards increased wound and chest infection was expected and seen, but was not significant. Compliance with postoperative chemotherapy, a potential surrogate marker for surgical morbidity, was not reduced in the preoperative group. Diarrhoea and skin rash were lower than previously reported with combination oxaliplatin, fluoropyrimidine, and EGFR antibody therapy,^17^ probably because only 31% received panitumumab—the panitumumab comparison is still masked—and also because of the short (6-week) duration preoperative treatment.

Another key finding is that a high-risk cohort suitable for combination chemotherapy can be identified by radiological assessment across multiple sites. The risk of exposing patients with early stage (pT2) disease to inappropriate chemotherapy was low, with only one patient of 51 (2%) being inaccurately upstaged, justifying the ongoing phase 3 trial. Chemotherapy for the 12% with pT3 tumours without additional risk factors is not unreasonable in view of the established efficacy of chemotherapy in stage 2 disease.^34^ In a parallel audit,^35^ 93% of patients with radiologically staged T3 tumours with less than 5 mm invasion of the muscularis propria were found to have coexisting high-risk pathological features that justify chemotherapy and the eligibility criteria for the full FOxTROT study have been amended accordingly. As radiological staging of primary colon cancer is underdeveloped, a series of workshops—attended by 200 gastrointestinal radiologists—were held to standardise radiological selection criteria and similar training would be advisable in other neoadjuvant trials.

The population studied in FOxTROT seemed typical of those who might be considered for neoadjuvant therapy. The median age of 63 years is 10 years younger than the colon cancer population as a whole, but typical for trials of adjuvant chemotherapy. The distribution of tumours in the study largely reflects that of colonic tumours in the population, other than fewer tumours from the rectosigmoid region, probably because of consideration for radiotherapy, an exclusion criterion. The fairly high (20%) resection margin involvement in control patients is probably explained by more rigorous histopathological assessment and by selection of high-risk patients.

Delivery of *KRAS* mutation analysis across 35 UK sites was another challenge, since delay in treatment would preclude clinical use. Our 9-day median time from consent to *KRAS* testing to randomisation for panitumumab was achieved with a two-stage consent process and, importantly, a histopathology principal investigator at every site who helped to ensure that the biopsy sample was released for analysis promptly. Another concern that testing of small biopsies would be inaccurate also proved unfounded, with an assay failure rate of only 4%, and the concordance between *KRAS* mutation assays of matched samples from the multiple biopsies and resected tumour was 99%,^36^ showing the reliability of DNA-based assays of colon cancer biopsies.

A particular strength of neoadjuvant over metastatic studies is that pre-treatment and post-treatment tissue samples are available to assess markers of tumour response. If validated as a surrogate of long-term oncological outcome, regression grading provides the most statistically sensitive measure of chemosensitivity to allow identification of predictive molecular markers and, potentially, to guide postoperative adjuvant treatment. Radiological assessment after preoperative therapy provided supportive evidence for real downstaging and could also be useful in assessment of primary tumour response and the potential value of extending preoperative therapy.

In summary, we have shown that patients with locally advanced, but resectable, colon cancer can be appropriately selected for neoadjuvant chemotherapy with CT scanning, can be molecularly stratified preoperatively, and can safely undergo preoperative chemotherapy followed by colonic resectional surgery, without incurring significant perioperative morbidity. We have also shown significant downstaging of primary tumours, including fewer incomplete resections and reduced apical lymph node metastases, after only 6 weeks of combination therapy. On the basis of these promising findings from the pilot phase, proceeding to the ongoing FOxTROT phase 3 study is appropriate; we aim to enrol at least a further 900 patients. If preoperative therapy results in fewer recurrences, as well as tumour downstaging, the established pathway of surgery then chemotherapy in the management of colon cancer could potentially change.
