# Supplementary material for: Feasibility of preoperative chemotherapy for locally advanced, operable colon cancer: the pilot phase of a randomised controlled trial
Source: Lancet Oncol. 2012 Nov;13(11):1152–60. doi: 10.1016/S1470-2045(12)70348-0 (PMC3488188; doi:10.1016/S1470-2045(12)70348-0)
Supplement: Supplementary appendix [file mmc1.pdf]

THE LANCET Oncology

Supplementary appendix

This appendix formed part of the original submission and has been peer reviewed. We post it as supplied by the authors.

Supplement to: FOxTROT Collaborative Group. Feasibility of preoperative chemotherapy for locally advanced, operable colon cancer: the pilot phase of a randomised controlled trial. *Lancet Oncol* 2012; published online Sept 25, 2012. http://dx.doi.org/10.1016/S1470-2045(12)70348-0.

Appendix Table and Figures Legends

Appendix figure 1. Response rates in 13 trials of chemotherapy with or without EGFR-directed monoclonal antibody therapies in *K-RAS*-wildtype metastatic colorectal cancer stratified by previous chemotherapy exposure.

Appendix figure 2. KRAS-testing flow-chart

Appendix table 1. Toxicity experienced in each course of chemotherapy in the pre- plus post-operative chemotherapy group compared with the postoperative chemotherapy group

Appendix figure 1: Response rates in 13 trials of chemotherapy with or without EGFR–directed monoclonal antibody therapies in KRAS–wildtype* metastatic colorectal cancer stratified by previous chemotherapy exposure.

Trial Name (reference)		Responses/Patients mAb	Responses/Patients Control	Statistics (O–E)	Statistics Var.	O.R. & 95% CI (Control : mAb)
**Progressive Disease: Best Supportive Care ± mAb:**						
Multinational (18)	BSC ± Cetuximab	15/117 (12.8%)	1/113 (0.9%)	6·9	3·7	6.26 (2.27, 17.25)
NCIC CO17 (19,20)	BSC ± Panitumumab	21/124 (16.9%)	0/119 (0.0%)	10·3	4·8	8.48 (3.47, 20.72)
**Subtotal:**		**36/241 (14.9%)**	**1/232 (0.4%)**	**17·1**	**8·6**	**7.42 (3.80, 14.51) 2P < 0·00001**
Test for heterogeneity between subgroups: $\chi^2_1 = 0{\cdot}2$; P = 0·66						
**Second–line chemotherapy ± mAb:**						
2005–181 (21)	FOLFIRI ± Panitumumab	104/297 (35.0%)	29/285 (10.2%)	36·1	25·7	4.08 (2.77, 6.01)
PICCOLO (22)	IRI ± Panitumumab	78/230 (33.9%)	28/230 (12.2%)	25·0	20·4	3.40 (2.20, 5.24)
EPIC (*All KRAS) (23)	IRI ± Cetuximab	106/648 (16.4%)	27/650 (4.2%)	39·6	29·9	3.76 (2.63, 5.39)
**Subtotal:**		**288/1175 (24.5%)**	**84/1165 (7.2%)**	**100·7**	**76·0**	**3.76 (3.01, 4.71) 2P < 0·00001**
Test for heterogeneity between trials: $\chi^2_2 = 0{\cdot}4$; P = 0·83						
**First–line chemotherapy ± mAb:**						
COIN (17)	OxMdG/OxCap ± Cetuximab	232/362 (64.1%)	209/367 (56.9%)	13·0	43·6	1.35 (1.00, 1.81)
CRYSTAL (24)	FOLFIRI ± Cetuximab	181/316 (57.3%)	139/350 (39.7%)	29·2	41·5	2.02 (1.49, 2.74)
OPUS (25)	FOLFOX ± Cetuximab	47/82 (57.3%)	33/97 (34.0%)	10·4	11·1	2.55 (1.41, 4.60)
PRIME (26)	FOLFOX ± Panitumumab	179/325 (55.1%)	159/331 (48.0%)	11·6	41·0	1.33 (0.98, 1.80)
PACCE (27)	FOX/FIRI + bev ± Panitumumab	131/258 (50.8%)	142/261 (54.4%)	–4·7	32·4	0.86 (0.61, 1.22)
CAIRO2 (28)	OxCap + bev ± Cetuximab	97/158 (61.4%)	78/156 (50.0%)	8·9	19·4	1.58 (1.02, 2.47)
SAKK (29)	OxCap ± Cetuximab	15/37 (40.5%)	5/37 (13.5%)	5·0	3·7	3.86 (1.39, 10.70)
NORDIC (30)	FLOX ± Cetuximab	45/97 (46.4%)	46/97 (47.4%)	–0·5	12·1	0.96 (0.55, 1.68)
**Subtotal:**		**927/1635 (56.7%)**	**811/1696 (47.8%)**	**72·8**	**204·9**	**1.43 (1.24, 1.64) 2P < 0·00001**
Test for heterogeneity between trials: $\chi^2_7 = 23{\cdot}0$; P = 0·002						
**Total:**		**1251/3051 (41.0%)**	**896/3093 (29.0%)**	**190·7**	**289·4**	**1.93 (1.72, 2.17)**

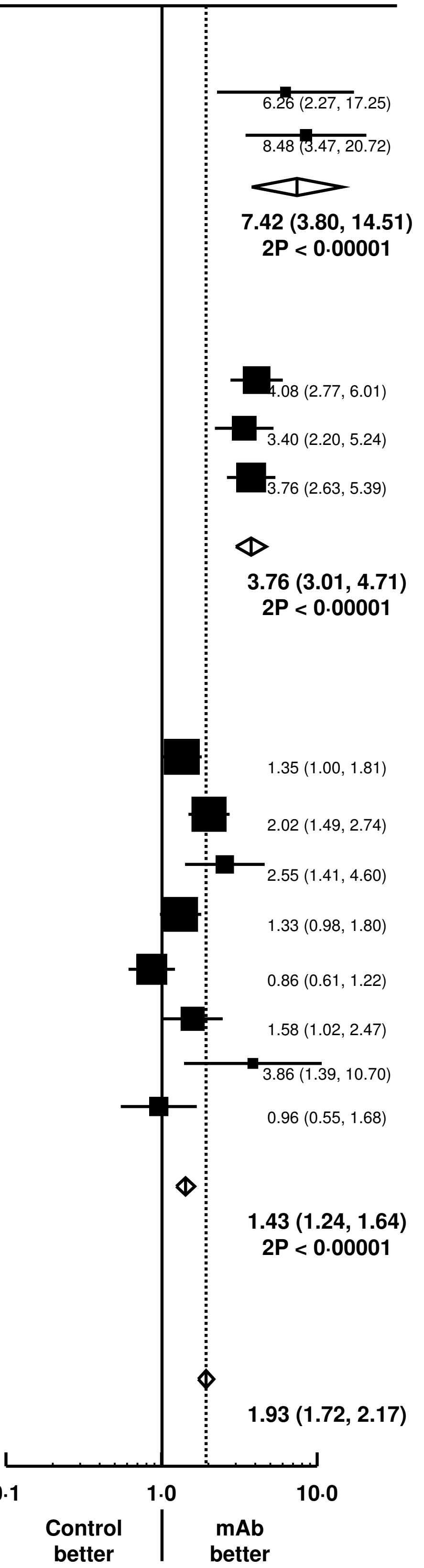

Effect 2P < 0·00001

Test for heterogeneity (13 groups): $\chi^2_{12} = 91{\cdot}7$; P < 0·00001

Test for heterogeneity between subtotals: $\chi^2_2 = 68{\cdot}1$; P < 0·00001

*KRAS–status was not available for EPIC study

Abbreviations: O.R.: Odds Ratio, O–E: Observed minus Expected numbers of events, Var: variance of O–E, BSC: Best Supportive Care, mAb: monoclonal antibody, bev: bevacizumab

Appendix figure 2. KRAS-testing flow-chart

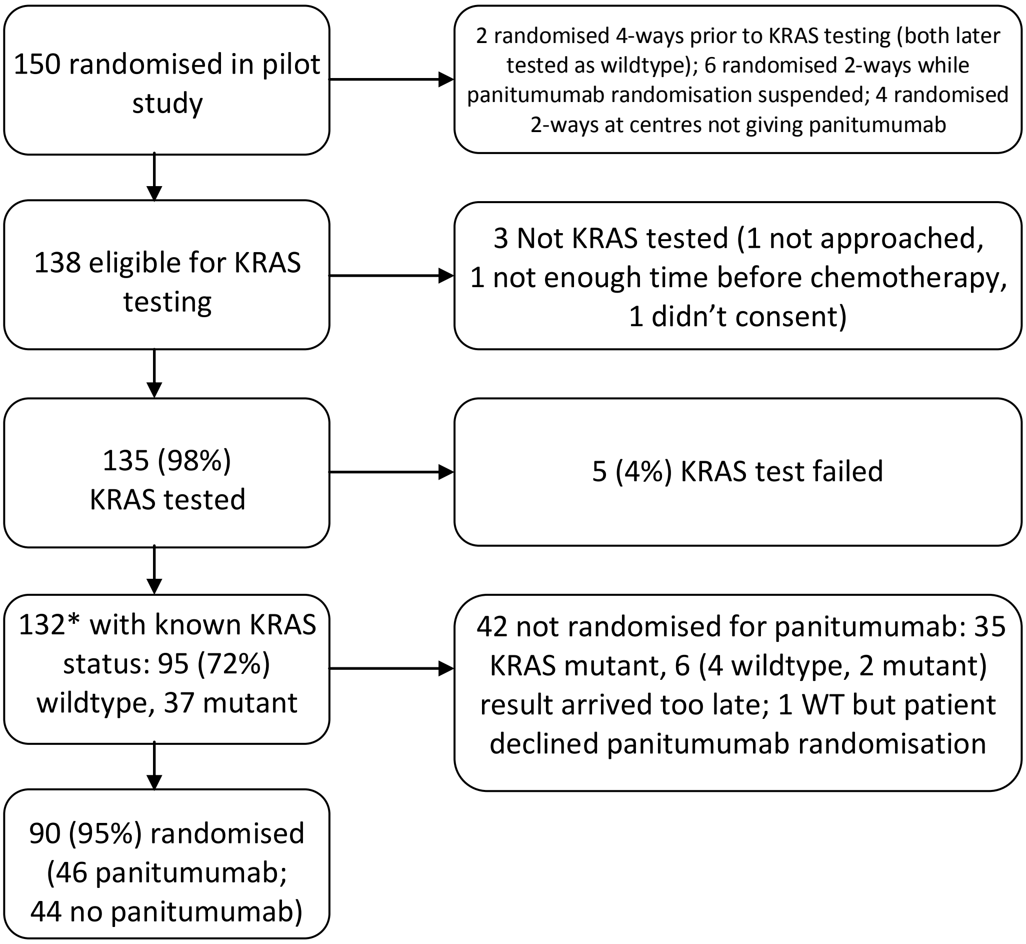

* includes 2 randomised 4-ways prior to KRAS testing (both later tested as wildtype)

Appendix table 1. Toxicity experienced in each course of chemotherapy in the pre- plus post-operative chemotherapy group compared with the postoperative chemotherapy group

**Patients starting course 1**	**Pre plus Post (n = 94[¥])**		**Post Only (n = 39)**		**p-value any toxicity**
**Toxicity grade*:**	**Any grade**	**Grade 3+**	**Any grade**	**Grade 3+**	
**Haematological**	**49 (52%)**	**19 (20%)**	**18 (46%)**	**5 (13%)**	**0.53**
Platelets	11	-	3	-	0.49
Haemoglobin	36	-	8	-	0.05
WBC	20	8	6	-	0.44
Neutrophils	30	19	10	5	0.47
**Skin**	**35 (37%)**	**7 (7%)**	**21 (54%)**	**3 (8%)**	**0.08**
Alopecia	5	-	5	-	0.14
Nail changes	3	-	2	-	0.59
Skin rash	26	7	13	3	0.51
Hand/foot syndrome	7	-	7	-	0.07
**Gastrointestinal**	**68 (72%)**	**7 (7%)**	**29 (74%)**	**4 (10%)**	**0.81**
Anorexia	24	4	6	-	0.20
Diarrhoea	27	5	22	3	0.003
Stomatitis	38	-	10	1	0.11
Nausea	34	1	19	-	0.18
Vomiting	18	2	2	-	0.04
**Pain**	**28 (30%)**	**5 (5%)**	**11 (28%)**	**2 (5%)**	**0.86**
Pain	27	5	11	2	0.95
Vein pain	3	-	-	-	0.27
**Lethargy**	**65 (69%)**	**6 (6%)**	**20 (51%)**	**1 (3%)**	**0.06**
**Peripheral neuropathy**	**47 (50%)**	**-**	**22 (56%)**	**-**	**0.50**
**Any category**	**90 (96%)**	**32 (34%)**	**36 (92%)**	**12 (31%)**	**0.42**

*Toxicity grading used the Common Terminology Criteria for Adverse Events v3.0

[¥] One patient provided chemotherapy treatment information but did not complete a toxicity assessment

Patients starting course 2	Pre plus Post (n = 81)		Post Only (n = 38)		p-value any toxicity
	Any grade	Grade 3+	Any grade	Grade 3+	
**Haematological**	**41 (51%)**	**9 (11%)**	**21 (55%)**	**4 (11%)**	**0.64**
Platelets	17	-	11	-	0.34
Haemoglobin	26	2	10	-	0.52
WBC	15	2	8	-	0.75
Neutrophils	20	7	13	4	0.28
**Skin**	**26 (32%)**	**-**	**15 (39%)**	**2 (5%)**	**0.43**
Alopecia	10	-	9	-	0.11
Nail changes	2	-	5	-	0.02
Skin rash	8	-	7	2	0.19
Hand/foot syndrome	9	-	2	-	0.31
**Gastrointestinal**	**49 (60%)**	**5 (6%)**	**31 (82%)**	**2 (5%)**	**0.02**
Anorexia	12	1	6	-	0.89
Diarrhoea	35	3	20	1	0.34
Stomatitis	48	4	24	-	0.69
Nausea	24	1	14	-	0.43
Vomiting	4	-	2	-	0.94
**Pain**	**22 (27%)**	**1 (1%)**	**10 (26%)**	**-**	**0.92**
Pain	21	1	8	-	0.57
Vein pain	1	-	2	-	0.19
**Lethargy**	**48 (59%)**	**4 (5%)**	**24 (63%)**	**-**	**0.69**
**Peripheral neuropathy**	**55 (68%)**	**1 (1%)**	**29 (76%)**	**2 (5%)**	**0.35**
**Any category**	**77 (95%)**	**14 (17%)**	**37 (97%)**	**9 (24%)**	**0.56**

**Patients starting course 3**	**Pre plus Post (n = 75)**		**Post Only (n = 34)**		**p-value any toxicity**
	**Any grade**	**Grade 3+**	**Any grade**	**Grade 3+**	
**Haematological**	**42 (56%)**	**6 (8%)**	**20 (59%)**	**4 (12%)**	**0.78**
Platelets	27	1	8	-	0.20
Haemoglobin	26	1	8	-	0.25
WBC	11	1	6	-	0.69
Neutrophils	23	6	15	4	0.17
**Skin**	**25 (33%)**	**1 (1%)**	**9 (26%)**	**-**	**0.48**
Alopecia	10	-	5	-	0.85
Nail changes	5	-	3	-	0.69
Skin rash	4	-	3	-	0.49
Hand/foot syndrome	15	1	2	-	0.06
**Gastrointestinal**	**48 (64%)**	**7 (9%)**	**23 (68%)**	**2 (6%)**	**0.71**
Anorexia	9	-	6	-	0.43
Diarrhoea	30	6	18	1	0.21
Stomatitis	25	2	11	1	0.92
Nausea	24	1	11	-	0.97
Vomiting	3	-	3	-	0.31
**Pain**	**17 (23%)**	**1 (1%)**	**2 (6%)**	**1 (3%)**	**0.03**
Pain	17	1	2	1	0.03
Vein pain	-	-	-	-	
**Lethargy**	**50 (67%)**	**3 (4%)**	**21 (62%)**	**2 (6%)**	**0.62**
**Peripheral neuropathy**	**48 (64%)**	**2 (3%)**	**30 (88%)**	**1 (3%)**	**0.01**
**Any category**	**74 (99%)**	**18 (24%)**	**33 (97%)**	**8 (24%)**	**0.56**

**Patients starting course 4**	**Pre plus Post (n = 73)**		**Post Only (n = 31)**		**p-value any toxicity**
	**Any grade**	**Grade 3+**	**Any grade**	**Grade 3+**	
**Haematological**	**39 (53%)**	**5 (7%)**	**9 (29%)**	**3 (10%)**	**0.02**
Platelets	26	1	5	1	0.05
Haemoglobin	22	-	4	-	0.07
WBC	15	-	2	-	0.08
Neutrophils	22	4	5	2	0.14
**Skin**	**21 (29%)**	**-**	**10 (32%)**	**-**	**0.72**
Alopecia	8	-	4	-	0.78
Nail changes	8	-	3	-	0.85
Skin rash	7	-	1	-	0.27
Hand/foot syndrome	10	-	5	-	0.75
**Gastrointestinal**	**37 (51%)**	**2 (3%)**	**14 (45%)**	**1 (3%)**	**0.61**
Anorexia	8	-	1	-	0.20
Diarrhoea	18	2	9	1	0.64
Stomatitis	18	-	8	-	0.90
Nausea	15	-	4	-	0.36
Vomiting	1	-	1	-	0.53
**Pain**	**8 (11%)**	**-**	**4 (13%)**	**-**	**0.78**
Pain	8	-	4	-	0.78
Vein pain	-	-	-	-	
**Lethargy**	**43 (59%)**	**3 (4%)**	**12 (39%)**	**-**	**0.06**
**Peripheral neuropathy**	**38 (52%)**	**-**	**24 (77%)**	**3 (10%)**	**0.02**
**Any category**	**66 (90%)**	**9 (12%)**	**28 (90%)**	**7 (23%)**	**0.99**

**Over all courses**	**Pre plus Post (n = 95)**		**Post Only (n = 39)**		**p-value any toxicity**
	**Any grade**	**Grade 3+**	**Any grade**	**Grade 3+**	
**Haematological**	**71 (75%)**	**28 (29%)**	**25 (64%)**	**11 (28%)**	**0.22**
Platelets	41	2	12	1	0.18
Haemoglobin	51	2	13	-	0.03
WBC	38	8	11	-	0.20
Neutrophils	51	28	21	10	0.99
**Skin**	**56 (59%)**	**8 (8%)**	**27 (69%)**	**3 (8%)**	**0.27**
Alopecia	15	-	9	-	0.32
Nail changes	10	-	6	-	0.43
Skin rash	32	7	15	3	0.60
Hand/foot syndrome	22	1	11	-	0.54
**Gastrointestinal**	**82 (86%)**	**18 (19%)**	**35 (90%)**	**8 (21%)**	**0.59**
Anorexia	38	5	10	-	0.12
Diarrhoea	54	14	31	5	0.01
Stomatitis	52	2	23	3	0.66
Nausea	56	3	23	-	1.00
Vomiting	24	2	5	-	0.11
**Pain**	**45 (47%)**	**6 (6%)**	**18 (46%)**	**2 (5%)**	**0.90**
Pain	44	6	18	2	0.99
Vein pain	3	-	2	-	0.59
**Lethargy**	**85 (89%)**	**13 (14%)**	**31 (79%)**	**3 (8%)**	**0.13**
**Peripheral neuropathy**	**77 (81%)**	**2 (2%)**	**37 (95%)**	**5 (13%)**	**0.04**
**Any category**	**94 (99%)**	**47 (49%)**	**39 (100%)**	**20 (51%)**	**0.96**
